# Effective doses of ciprofol combined with alfentanil in inhibiting responses to gastroscope insertion, a prospective, single-arm, single-center study

**DOI:** 10.1186/s12871-023-02387-4

**Published:** 2024-01-02

**Authors:** Xiaoru Wu, Min Liao, Xingzhou Lin, Jianing Hu, Tangyuanmeng Zhao, Hu Sun

**Affiliations:** 1 Department of Anesthesiology, The Second Affiliated Hospital of Hainan Medical University, Hai Kou, 570311 China; 2 Department of Anesthesiology, West China Hospital, Sichuan University, Chengdu, 610041 China

**Keywords:** Ciprofol, Alfentanil, Gastroscopy, Effective dose, Dixon’s up-and-down method

## Abstract

**Background:**

Ciprofol is a novel intravenous sedative and anesthetic. Studies have shown that it features a rapid onset of action, a fast recovery time, slight inhibition of respiratory and cardiovascular functions, and a low incidence of adverse reactions. This study aims to explore the median effective dose (ED_50_) and the 95% effective dose (ED_95_) of ciprofol in inhibiting responses to gastroscope insertion when combined with a low dose of alfentanil, and to evaluate its safety, to provide a reference for the rational use of ciprofol in clinical practices.

**Methods:**

We included 25 patients aged 18–64 years of either sex who underwent gastroscopy under intravenous general anesthesia, with a Body Mass Index (BMI) 18–28 kg/m^2^, and an American Society of Anesthesiologists (ASA) grade I or II. In this study, the dose-finding strategy of ciprofol followed a modified Dixon’s up-and-down method with an initial dose of 0.30 mg/kg and an increment of 0.02 mg/kg. Ciprofol was administered after intravenous injection of 7 µg/kg of alfentanil, and 2 min later a gastroscope was inserted. When the insertion response of one participant was positive (including body movement, coughing, and eye opening), an escalation of 0.02 mg/kg would be given to the next participant; otherwise, a de-escalation of 0.02 mg/kg would be administered. The study was terminated when negative response and positive response alternated 8 times. A Probit model was used to calculate the ED_50_ and ED_95_ of ciprofol in inhibiting responses to gastroscope insertion when combined with alfentanil. Patients’ recovery time, discharge time, vital signs and occurrence of adverse reactions were recorded.

**Results:**

The ED_50_ of single-dose intravenous ciprofol injection with 7 µg/kg of alfentanil in inhibiting gastroscope insertion responses was 0.217 mg/kg, and the ED_95_ was 0.247 mg/kg. Patients’ recovery time and discharge time were 11.04 ± 1.49 min and 9.64 ± 2.38 min, respectively. The overall incidence of adverse reactions was 12%.

**Conclusion:**

The ED_50_ of ciprofol combined with 7 µg/kg of alfentanil in inhibiting gastroscope insertion responses was 0.217 mg/kg, and the ED_95_ was 0.247 mg/kg. Ciprofol showed a low incidence of anesthesia-related adverse events.

**Trial registration:**

http://www.chictr.org.cn (ChiCTR2200061727).

**Supplementary Information:**

The online version contains supplementary material available at 10.1186/s12871-023-02387-4.

## Background

As the gold standard for diagnosing gastrointestinal diseases, digestive endoscopy has received considerable attention. Compared with ordinary gastroscopy, gastroscopy with anesthesia brings great benefits [1]. Despite all its advantages, painless gastroscopy possesses disadvantages that could not be ignored. For example, excessive sedation may delay patient recovery, prolong hospitalization, increase the overall cost of endoscopy, and increase the risk of respiratory and cardiovascular complications [1]. Thus, it is particularly important to explore an optimal solution that can improve patient comfort and reduce adverse reactions caused by anesthetics during endoscopic exams.

Propofol is currently one of the most used intravenous anesthetics for outpatient gastroenteroscopy. However, in clinical application, it has limitations of a narrow therapeutic window, dose-dependent inhibition of cardiovascular and respiratory functions and a high incidence of injection site pain, which seriously affects patient satisfaction [2]. Ciprofol is a novel intravenous sedative and anesthetic with a chemical structure similar to propofol. It has a rapid onset of action, a fast recovery, a high potency, a wide therapeutic window, slight inhibitory effects on respiratory and cardiovascular functions, and a low incidence of adverse reactions [3–8]. Such advantages render it more suitable for outpatient surgeries [9]. There has been confirmed that an intravenous anesthetic combined with a low-dose analgesic enjoys significant merits in anesthesia for gastroscopy [10], and alfentanil, among all analgesics, demonstrates sound effectiveness in such scenario [11].

Due to the pharmacological characteristics of ciprofol and alfentanil, combining the two agents may be the optimal anesthesia for gastroscopy. However, there is currently no relevant report on the recommended dose of ciprofol combined with alfentanil in such scenario. Chen et al. [12] found 7 µg/kg of alfentanil combined with an intravenous anesthetic to be the best analgesic strategy for gastroscopy anesthesia. We conducted a pre-test with reference to the recommended dose of Chen et al. and found that alfentanil 7 µg/kg was effective for gastroscopy anesthesia, so we combined ciprofol with 7 µg/kg of alfentanil to determine its effective doses.

On the basis of previous preliminary experiments, this study aims to explore the ED_50_ and ED_95_ of ciprofol in inhibiting responses to gastroscope insertion when combined with a low dose of alfentanil, and to evaluate its safety during endoscopy, to provide a reference for the rational use of ciprofol in clinical practices.

## Methods

### Ethics and registration

This study has been approved by the Medical Ethics Committee of the Second Affiliated Hospital of Hainan Medical University (approval number: LW202051), and registered at http://www.chictr.org.cn (registration number: ChiCTR2200061727, 1/7/2021). The research protocol was carried out under the guidance of relevant guidelines. All patients or their families signed informed consent.

### Patient selection

This study is a prospective, single-blind, single-center research carried out in the Second Affiliated Hospital of Hainan Medical University. Participants underwent painless gastroscopy in our hospital from July to August 2022. To ensure homogeneity, the inclusion and exclusion criteria of this study are as follows. Patients included were those aged 18–64 years of either sex, with a Body Mass Index (BMI) 18–28 kg/m^2^, and American Society of Anesthesiologists (ASA) grade I or II. Exclusion criteria include presence of difficult airway, allergy to the anesthetic used, history of alcohol, sedative or analgesic abuse, mental illness, pregnant and lactating women. Patients who required manual control of ventilation or whose gastroscopy lasted more than 30 min were excluded.

### Study design

To ensure accurate dosing, we used 0.9% sodium chloride injection to dilute alfentanil (Yichang Renfu Pharmaceutical, China, 13S11041) to 50 µg/ml; ciprofol (Liaoning Hesco Pharmaceutical Co., Ltd., China, 20211104) was diluted to 1.25 mg/ml with 0.9% sodium chloride injection according to its Instructions for Use.

According to the American Society for Gastrointestinal Endoscopy (ASGE) guideline, all patients routinely fasted for solids for at least 6 h and fasted for water for at least 2 h [13]. Venous access was established when patients entered the endoscopy room. Patients lay on their left sides before being connected to a monitor (Bene View N15 OR monitor, Myriad Biomedical Electronics Co., Shenzhen, China) for continuous monitoring of electrocardiogram (ECG), non-invasive blood pressure (NIBP), saturation of peripheral oxygen (SpO_2_), respiratory rate (RR) and heart rate (HR). All indicators were measured three times, and the average of each was determined as the baseline value. 3-5 min before the start of gastroscopy, the patients were given nasal cannula for oxygenation (4–6 L/min) in a spontaneous breathing mode and continued until the end of the examination and were fully awake.

### Intervention and remedial measures

In this study, the dose-finding strategy of ciprofol followed a modified Dixon’s up-and-down method with an arithmetic sequence. The modified Dixon’s up-and-down design is a classic method to explore the median effective dose of an agent where the dosage of a participant is determined based on the response of the previous one. The biggest advantage of this method is that it only needs 1/4 to1/3 of the sample size of the traditional method (usually ≥ 6 pairs of negative-positive responses are recommended) to draw the same reliable conclusions [14]. According to relevant literature and preliminary experiments, the initial dose of ciprofol was 0.30 mg/kg with an increment of 0.02 mg/kg [5]. Alfentanil was injected intravenously at 7 µg/kg (administration duration 30 s), followed by intravenous administration of ciprofol at a uniform speed for 30 s, and a gastroscope was inserted 2 min after the end of dosing, that is, when the plasma concentration of ciprofol reached its peak [9]. When the insertion response of one participant was positive, an escalation of 0.02 mg/kg would be given to the next participant; otherwise, a de-escalation of 0.02 mg/kg would be administered. The study was terminated when negative response and positive response alternated 8 times. The definition of a positive response referred to reactions such as body movement, coughing, and eye opening during gastroscopy insertion [15]. All gastroscopic exams were performed by endoscopists with rich experience and mature technique who had been engaged in endoscopy for more than 3 years. All anesthesia operations were completed by the same anesthesiologist, and another anesthesiologist who was not aware of the research oversaw data documentation.

The depth of sedation was evaluated using the Modified Observer’s Assessment of Alert Score (MOAA/S) (see Additional Supplementary Table 1) after each successful gastroscope insertion and before the completion of the endoscopic exam. Studies have shown that a MOAA/S ≤ 2 means that patients meet all requirements of endoscopy [16]. For patients showing positive responses during endoscope insertion or the MOAA/S score was above 2 points anytime during the exam, ciprofol 0.05–0.2 mg/kg would be injected intravenously to deepen the anesthesia (administration duration of 10 s, each additional dose should be given with an interval ≥ 2 min, and no more than 5 additions per 15 min) [9], until the completion of endoscopy. In case of bradycardia where HR < 50 beats/min during the exam, 0.5 mg of atropine would be administered intravenously. For hypotension (30% drop in blood pressure from preoperative baseline), ephedrine would be injected intravenously to maintain blood pressure. When patients’ SpO_2_ dropped below 90%, oxygenation would be provided by either jaw lift or pressurized mask ventilation, and an oropharyngeal airway would be established when necessary. Depending on the degree of hypoxia in the patient, the decision to terminate the test is made.

### Indicators

#### Primary indicators

The ED_50_ and ED_95_ of ciprofol in inhibiting responses to gastroscope insertion when combined with 7 µg/kg of alfentanil.

#### Secondary indicators

MAP, HR and SpO_2_ at T0 (patients entering the endoscopy room), T1 (1 min after ciprofol injection), T2 (gastroscope inserting into the pharyngeal cavity), T3 (immediately after gastroscope withdrawal), and T4 (patients were fully awake); recovery time, discharge time; occurrence of hypotension (30% or more drop in blood pressure from preoperative baseline), SpO_2_ below 90%, intravenous injection site pain, muscle stiffness, nausea and vomiting, intraoperative awareness, restlessness during recovery, delayed recovery, and other adverse reactions.

Recovery time was defined as the time from the last intravenous injection of ciprofol to two consecutive MOAA/S ≥ 4 evaluations (assessed every 1 min). The modified Aldrete score (see Additional Supplementary Table 2) is used to evaluate whether the patient met the criteria for discharge [17]. Usually, patients with an Aldrete score ≥ 9 can be discharged. The discharge time was defined as the time from complete recovery to meeting all discharge requirements. Intravenous injection pain was defined as avoidance movements in the ipsilateral upper extremity or complaints of pain at the injection site during ciprofol administration. The Richmond Agitation-Sedation Scale (RASS) (see Additional Supplementary Table 3) was applied to evaluate the existence of agitation during the recovery period. A RASS score 1–4 points translates into positive agitation [18].

### Sample size

The modified Dixon’s up and down method requires at least 6 pairs of negative-positive responses to calculate a reliable ED_50_ [14]. This study was stopped when negative and positive responses alternated 8 times. A total of 25 patients were included, and the sample size was reliable.

### Statistical analysis

Statistical analysis was performed using SPSS 25.0 statistical software (SPSS Inc., Chicago, IL, U.S.A.). The Kolmogorov-Smirnov test was used for normal distribution examination. Normally distributed indicators were described as mean ± standard deviation. Categorical variables were expressed as percentages (%). Data at different time points were analyzed by repeated measures ANOVA. ED_50_, ED_95_ and their corresponding 95% confidence intervals (CI) were calculated using the Probit method; based on the probability model, the fitting equation was derived and the dose-effect curve was drawn. The up-and-down dose-finding curve and the dose-effect fitting curve were drawn using Microsoft Excel 2016 software. A *P* value < 0.05 is statistically significant.

## Results

### Patient characteristics

**Fig. 1:**
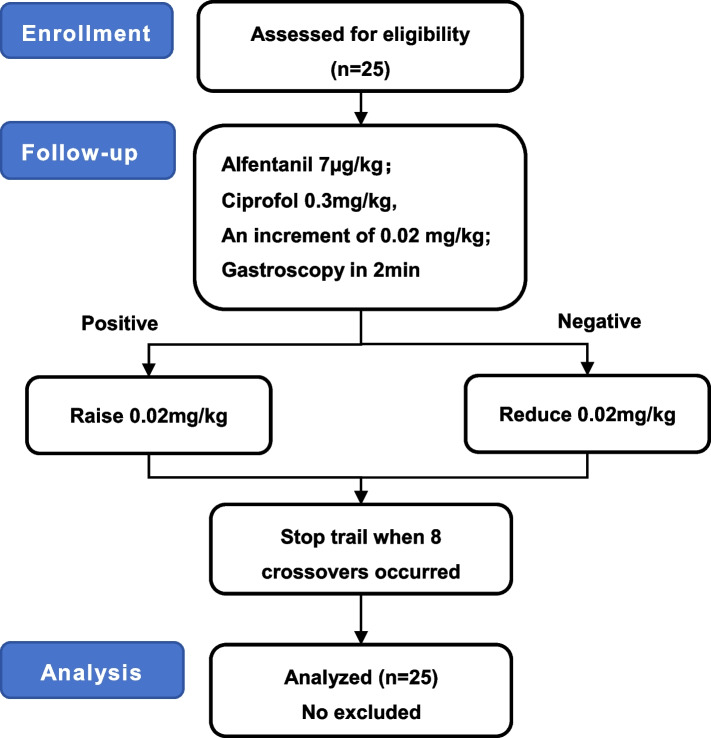
Participant flow diagram (Dixon’s up and down method)

A total of 25 cases were included in this study. The participant flow diagram is shown in Fig. 1. Demographic characteristics of these patients are shown in Table 1, and no cases were excluded.

**Table 1:** Demographic characteristics of patients

**Characteristics**	***n* = 25**
Sex (men/women)	14/11
Age (year)	45.08 ± 15.10
BMI (kg/m^2^)	22.78 ± 2.95
ASA grade (I/II) (%)	18 (72%)/7 (28%)

**Fig. 2:**
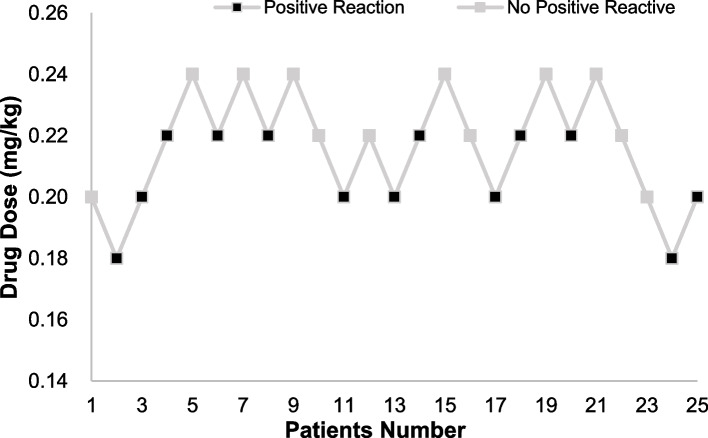
Dixon’s up‑and‑down method

**Fig. 3:**
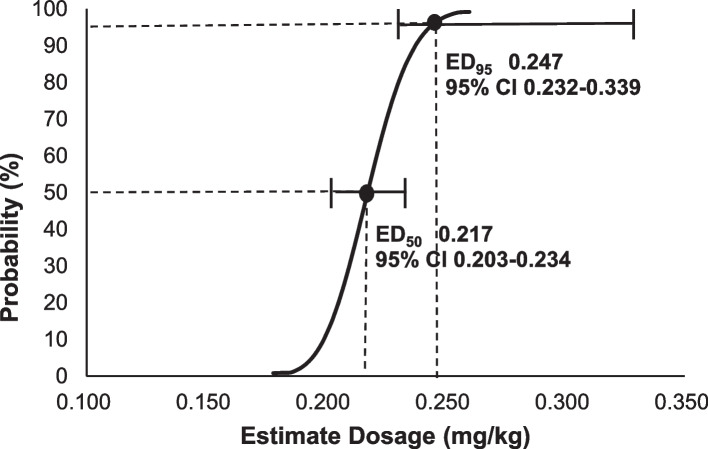
The dose–effect curve, Probit (*p*) = -11.927 + 54.896X. (The covariate X, which represents the estimate dosage, is converted using a logarithm with base 10). Horizontal bars denote 95% CI for ED_50_ and ED_95_

Of the 25 patients, 13 had positive responses and 12 had negative responses. Results of the up-and-down experiment are shown in Fig. 2. The ED_50_ of ciprofol combined with 7 μg/kg of alfentanil in inhibiting responses to gastroscope insertion was 0.217 mg/kg (95% CI: 0.203–0.234 mg/kg), and the ED_95_ was 0.247 mg/kg (95% CI: 0.232–0.339 mg/kg). The dose-effect fitting curve is shown in Fig. 3.

Changes in patients’ vital signs are shown in Table 2. Compared with T0, MAP significantly decreased at T1, T2, T3, and T4 (*P* < 0.001); HR slowed down at T2 (*P* < 0.05).

**Table 2:** Patients’ vital signs at different time points (*n* = 25)

**Indicators**	**T0**	**T1**	**T2**	**T3**	**T4**
MAP(mmHg)	84.97 ± 10.32	68.92 ± 10.24^b^	67.72 ± 11.85^b^	68.75 ± 10.19^b^	78.29 ± 10.65^b^
HR(bpm)	75.12 ± 17.90	70.24 ± 13.72	68.28 ± 12.04^a^	67.92 ± 10.26	70.36 ± 10.55
SpO_2_ (%)	99.32 ± 0.69	99.04 ± 0.79	98.28 ± 2.09	98.92 ± 0.76	99.12 ± 0.73

Compared to T0, ^a^*P* < 0.05 and ^b^*P* < 0.001

The recovery time was 11.04 ± 1.49 min, and the discharge time was 9.64 ± 2.38 min. Among all patients, 2 (8%) had hypotension, and 1 (4%) had SpO_2_ < 90% (the overall incidence of adverse reaction was 12%), and they returned to normal quickly after ephedrine injection to boost blood pressure and jaw lift for oxygenation, respectively. No anesthesia-related adverse events occurred (such as bradycardia, injection pain, muscle stiffness, nausea and vomiting, intraoperative awareness, restlessness during recovery, and delayed recovery).

## Discussion

Results showed that the ED_50_ of ciprofol injection with 7 µg/kg of alfentanil in inhibiting gastroscope insertion responses was 0.217 mg/kg, and the ED_95_ was 0.247 mg/kg. Huang et al. [19] argued that a sound anesthetic effect could be induced with 0.6 mg/kg of ciprofol when used alone in gastroscopy. In our study, the ED_95_ of ciprofol was only 1/3 to 1/2 of the abovementioned recommended dose, reflecting the advantage of combination strategy in reducing intravenous anesthetic dose, which is in line with expectations [10]. Besides, our ED_95_ is also lower than the reported recommendation of 0.4 mg/kg to meet all anesthesia requirements of gastroscopy when combined with 0.1 μg/kg of sufentanil [19], which can be partially explained by our small increment and accurate calculation. The use of different opioid analgesics in these studies which may lead to variations in the effective dose of ciprofol can also play a role.

The overall incidence of adverse reactions of the current study was 12%, significantly lower than the 19.3% and 16.1% reported by Huang [19] and Teng [5], respectively. Hypotension occurred in 8% of patients in this research and hypoxemia occurred in 4%, which were rapidly resolved after symptomatic treatment. It was consistent with the finding of Teng and Zhong. Teng [5] found that the incidence of ciprofol hypotension was lower than propofol (3.0% vs 10.0%), and Zhong [3] found that the incidence of ciprofol hypoxemia was lower than propofol (10.1% vs 2.9%). The above research illustrates that ciprofol may slightly impact the circulation and respiratory systems when used for endoscopic exams [5]. Ciprofol may cause circulatory and respiratory depression for the same reasons as propofol, both by inhibiting myocardial contraction or vascular tension and reducing tidal volume [20, 21], but its specific mechanism needs further exploration. And the mild inhibitory effect of ciprofol on the respiratory and circulatory systems may be related to its high potency, resulting in a lower dose [8]. The incidence of hypotension(8% vs 25%) and SpO_2_ < 90% (4% vs 6%) was lower in our study than Yi’s [22], which may be related to the fact that Yi’s study population was elderly, and it may also related to the fact that our study compounded alfentanil, which has a milder effect on respiratory circulation than sufentanil [11]. Although the incidence of adverse reactions in our study was low, both MAP and HR decreased after anesthesia induction. Thus, anesthesiologists need to strengthen perioperative monitoring. Generally, ciprofol combined with alfentanil is a relatively safe anesthesia strategy for gastroscopy.

Severe intravenous injection pain in the perioperative period could significantly aggravate patients’ tension and anxiety, directly or indirectly affect the stability of anesthesia induction, and seriously affect patients’ willingness to seek medical treatment and their experience of anesthesia [23]. In our study, none of the 25 patients experienced obvious pain from intravenous injection, which was consistent with the ciprofol’s phase III clinical trial [4]. That is, the incidence of ciprofol injection pain is significantly lower than that of propofol (4.4% vs 39.4%). The absence of obvious intravenous pain in this study could be explained by differences in the three-dimensional structure of ciprofol, dilution with normal saline, and early intravenous injection of short-acting analgesics [24–26].

The up-and-down design is a classic method to explore the median effective dose of an agent where the dosage of a participant is determined based on the response of the previous one. The tested dose is approaching the actual ED_50_ quickly, which not only saves manpower and time, but also limits the number of participants receiving the suboptimal regimen. In addition, the sample size required is only about 1/4 to 1/3 of the traditional method (usually ≥ 6 pairs of negative-positive responses are recommended) to draw equally reliable conclusions [14]. The more folds the more accurate the results are [27]. Therefore, in order to improve the reliability of the results, we finally decided to stop our study with 8 folds. In this study, an arithmetic increase/decrease was adopted after the initial dose of ciprofol was determined by preliminary experiments to ensure the accuracy of ciprofol dosing and reduce the difficulty of dispensing [28].

There are some advantages of our study. The greatest strength of our study is that we used a small sample size to explore the ED_50_ and ED_95_ of newly marketed clinical drugs for gastroscopy, and these precise values can provide a reference for the rational use of drugs in the clinic. Also, the analgesic we compounded was alfentanil, whose benefits in painless gastroscopy anesthesia are well documented [11].

Our study also has some limitations. First, the 95% CI of ED_50_ and ED_95_ in our study ranged widely, so further research is needed to determine the optimal dose of ciprofol for painless gastroscopy. Second, we only discussed the effective doses of ciprofol combined with alfentanil for the general population to inhibit gastroscope insertion responses, and age and gender had an impact on drug metabolism [29, 30]. Further studies are needed to explore effective doses for other populations. Third, the sample size of 25 patients, while adequate for the modified Dixon’s up-and-down method, is relatively small for generalizing the findings. And more, our findings to prevent response to insertion of gastroscope are limited to ciprofol plus alfentanil and cannot be extrapolated when ciprofol is administered alone or in combination with sedatives or other opioids.

## Conclusion

In summary, the ED_50_ of single-dose intravenous ciprofol injection combined with 7 µg/kg of alfentanil in inhibiting gastroscope insertion responses was 0.217 mg/kg, and the ED_95_ was 0.247 mg/kg. Ciprofol showed a low incidence of anesthesia-related adverse events.

### Supplementary Information

**Additional file 1: Table 1.** Modified Observer’s Assessment of Alertness/Sedation (MOAA/S) Scale. **Table 2.** The modified Aldrete score. **Table 3.** Richmond Agitation-Sedation Scale (RASS).

**Additional file 2.**

## Data Availability

The data that support the findings of this study are available from the corresponding author upon reasonable request.

## Abbreviations

ASA: American Society of Anesthesiologist
BMI: Body mass index
ECG: Electrocardiogram
NIBP: Noninvasive blood pressure
MAP: Mean arterial pressure
SpO_2_: Blood oxygen saturation
RR: Respiratory rate
HR: Heart rate
ASGE: American Society for Gastrointestinal Endoscopy
MOAA/S: Modified Observer’s Assessment of Alertness/Sedation
RASS: Richmond Agitation-Sedation Scale
