# Supplementary material for: Effective doses of ciprofol combined with alfentanil in inhibiting responses to gastroscope insertion, a prospective, single-arm, single-center study
Source: BMC Anesthesiol. 2024 Jan 2;24:2. doi: 10.1186/s12871-023-02387-4 (PMC10759617; doi:10.1186/s12871-023-02387-4)
Supplement: Supplementary file 1 — Additional file 1: Table 1. Modified Observer’s Assessment of Alertness/Sedation (MOAA/S) Scale. Table 2. The modified Aldrete score. Table 3. Richmond Agitation-Sedation Scale (RASS). [file 12871_2023_2387_MOESM1_ESM.docx]

**Additional Supplementary Table**

**Table 1 Modified Observer’s Assessment of Alertness/Sedation (MOAA/S) Scale**

| **MOAA/S Scale** | **Scale** |
| --- | --- |
| Does not respond to painful trapezius squeeze | 0 |
| Responds only after painful trapezius squeeze | 1 |
| Responds only after mild prodding or shaking | 2 |
| Responds only after name is called loudly and/or repeatedly | 3 |
| Lethargic response to name spoken in normal tone | 4 |
| Responds readily to name spoken in normal tone | 5 |

Scores were 4 to 5 for awake and 0 to 3 for sleep.

**Table 2 The modified Aldrete score**

| **Categories** | **Points** |
| --- | --- |
| Respiration |  |
| Able to breathe deeply and cough | 2 |
| Dyspnea or shallow breathing Apnea | 1 |
| Apnea | 0 |
| Oxygen saturation (SpO_2_) |  |
| Maintains > 92% on room air | 2 |
| Needs O_2_ inhalation to maintain O_2_ saturation ≧ 90% | 1 |
| SpO_2_ < 90% (with supplemental oxygen administered) | 0 |
| Consciousness level |  |
| Fully awake | 2 |
| Arousable upon calling | 1 |
| Not responding | 0 |
| Circulation |  |
| BP ± 20 mmHg (relative to pre-operation standard value) | 2 |
| BP ± 20–50 mmHg (relative to the pre-procedural standard value) | 1 |
| BP ± 50 mmHg (relative to the pre-procedural standard value) | 0 |
| Activity |  |
| Able to move the four extremities | 2 |
| Able to move two extremities | 1 |
| Cannot move the four extremities | 0 |

Discharge standards are satisfied if the score is ≥ 9.

BP: blood pressure

**Table 3 Richmond Agitation-Sedation Scale (RASS)**

| **Term** | **Description** | **Score** |
| --- | --- | --- |
| Combative | Overtly combative or violent; immediate danger to staff | +4 |
| Very agitation | Pulls on or removes tube(s) or catheter(s) or has aggressive behavior toward staff | +3 |
| Agitated | Frequent nonpurposeful movement or patient–ventilator dyssynchrony | +2 |
| Restless | Anxious or apprehensive but movements not aggressive or vigorous | +1 |
| Alert and calm |  | 0 |
| Drowsy | Not fully alert, but has sustained (more than 10 seconds) awakening, with eye contact, to voice | -1 |
| Light sedation | Briefly (less than 10 seconds) awakens with eye contact to voice | -2 |
| Moderate sedation | Any movement (but no eye contact) to voice | -3 |
| Deep sedation | No response to voice, but any movement to physical stimulation | -4 |
| Unarousable | No response to voice or physical stimulation | -5 |

RASS is a 10-point scale, with four levels of anxiety or agitation (+1 to +4 [combative]),

one level to denote a calm and alert state (0), and 5 levels of sedation (−1 to −5)

culminating in unarousable (−5).
